# Lymphopenia in thymectomized infants following congenital heart disease repair: implications for protective measures

**DOI:** 10.1186/s13019-026-04602-y

**Published:** 2026-08-17

**Authors:** Albraa Abulhamail, Abdulsalam O. Alsulami, Saad Samargandy, Heba Ekpal Mahboob, Badraa Abdulrahman Alahmadi, Khlod Ahmad Ywsef, Saddiq B. Habiballah, Mohammed Bin Jobah

**Affiliations:** 1 Pediatrics Department, King Abdulaziz University, Jeddah, Saudi Arabia; 2 Department of Preventive Medicine and Public Health, Faculty of Medicine, King Abdulaziz University, Jeddah, Saudi Arabia; 3 Faculty of Medicine, King Abdulaziz University, Jeddah, Saudi Arabia; 4 Department of Pediatrics, Faculty of Medicine Immunology unit, King Fahd Medical Research center, King Abdulaziz University, Jeddah, Saudi Arabia; 5 Department of Pediatrics, Pediatrics intensive care unit, Sulaiman Alhabib Hospital, Jeddah, Saudi Arabia

**Keywords:** Congenital heart disease, Thymectomy, Lymphopenia, Immune function, Pediatrics

## Abstract

**Background:**

Congenital heart disease (CHD) is a common birth defect frequently requiring surgical repair with thymectomy. While transient neonatal lymphopenia is usually benign, persistent lymphopenia may increase susceptibility to infections and immune dysregulation. This study examines the prevalence and longitudinal course of lymphopenia after thymectomy in infants with CHD, contributory factors, and infection risk.

**Method:**

This is a retrospective cohort study in which all children <2 years who underwent first congenital heart surgery with thymectomy between 2017–2022 for conotruncal defects were recruited. Baseline, post-operative, long-term lymphocyte counts, and episodes of infections for 3 years following the surgery were collected.

**Results:**

The data were collected from 95 patients with median age at the time of surgery 9.0 (IQR 5.0, 12.0) months and median weight 5.0 (1.0, 25.0) Kg. Tetralogy of Fallot (TOF) represented 53.68% of the sample. Prevalence of baseline lymphopenia was 11.6%, and within 1 week following the surgery increased to 54.7%. Being lymphopenic at baseline was associated with a decrement in lymphocyte count postoperatively by 1.2 × 10^9/L (*P* = 0.02) even after adjustment for failure to thrive.

**Conclusion:**

Immediate and long-term lymphopenia is highly prevalent post CHD surgical repair. Earlier age at the time of surgery, baseline lymphopenia, and being failure to thrive were significant predictors for lymphopenia post CHD surgical repair with total thymectomy. Subsequent lymphopenia not necessarily correlates with increase rate or severity of the infections. Pre-, post-operative and long-term lymphocyte count screening and clinical monitoring are warranted.

**Supplementary Information:**

The online version contains supplementary material available at https://doi.org/10.1186/s13019-026-04602-y.

## Introduction

Congenital heart diseases (CHD) are any structural or functional defect that happens to the heart or great vessels in the embryonic stage [1]. It is among the highest prevalent birth defect in Saudia Arabia with a reported prevalence of 8.7–12.3 per 1000 live births [2]. The ultimate treatment for 40% of the cases were surgical intervention with sternotomy and subsequent thymectomy [2]. With advancements in diagnostic and therapeutic techniques in this field, children with CHD fortunately have improved life expectancy, leading to a growing recognition of the long-term complications associated with cardiac surgery, particularly those of an immunological nature [3, 4]. 

Neonatal lymphopenia is considered a screening marker for inborn error of immunity [5]. Whilst transient lymphopenia of neonate is another entity that is usually not associated with long-term sequala, and often spontaneous recovery happens within months [6–8], persistence of lymphopenia is associated with more frequent and more severe infections [9]. In the context of surgical thymectomy, however, lymphopenia reflects a more fundamental disruption. The permanent loss of a primary lymphopoietic organ during a critical developmental window, leading to depletion of naïve T cells, restriction of T cell receptor repertoire diversity, and impaired central tolerance. These changes have been associated with recurrent infections, viral complications, and immune dysregulation, with an immunophenotypic profile that closely resembles that seen in 22q11.2 deletion syndrome, even in the absence of any underlying genetic abnormality [10, 11]. 

The relationship between CHD and lymphopenia, however, remains a subject of debate. While some research indicates that patients with CHD maintain normal lymphocyte counts before surgery [12], others have identified a predisposition for baseline lymphopenia in individuals with complex CHD, signaling a potentially poorer prognosis even before undergoing thymectomy [13]. Notably, conotruncal heart defects, accounting for 10–15% of all CHD cases, are drawing attention in the field of immunology due to their frequent association with syndromes known for their immunological sequelae, such as the 22q11.2 deletion syndrome [14]. Nonetheless, patients with non-syndromic conotruncal heart defects also display a higher risk of lymphopenia preceding thymectomy compared to their peers with non-conotruncal defects, albeit the difference was statistically insignificant. The relationship between conotruncal defects and lymphopenia may be related to the shared embryological origin between the conotruncus and the thymus [14]. Moreover, conotruncal anomalies have been shown to carry a disproportionately greater immunological burden compared to other CHD subtypes, with more pronounced reductions in T-helper cells, immunoglobulins, and complement levels even prior to surgical intervention — further underscoring the biological rationale for focusing on this specific population [15]. 

Thymus, a critical lymphoid organ, fosters the maturation and proliferation of T lymphocytes by providing an essential microenvironment for the development of the TCR repertoire and the establishment of central tolerance [12, 16]. Thymectomy, commonly performed during surgical correction of congenital heart defects to improve surgical field visibility, is increasingly recognized for its long-term immunological impact. Recent data suggest that children undergoing early thymectomy experience a significant and progressive loss of naïve lymphocyte populations, alongside a constrained T cell receptor Vβ repertoire, with the most pronounced decline occurring within the first three to six months post-surgery [12, 16]. The implications of early thymectomy—and potentially of CHD itself—extend beyond lymphocyte counts, affecting T cell functionality and leading to a heterogeneous immunological profile characterized by heightened inflammation, increased risk of infection, and autoimmunity [11, 17]. *Other reports highlighted the incidence of lymphopenia post-surgery*,* but clinically there were no significant increase in acquiring infections or prolonging the course of illness.* [18, 19]

At our tertiary center, which specializes in treating patients with complex heart defects, we are uniquely positioned to investigate the relationship between cardiac defects—particularly conotruncal defects—baseline lymphopenia, and the short- and long-term impacts of thymectomy on absolute lymphocyte count. This study represents the first of its kind in the Kingdom of Saudi Arabia to explore this specific topic. Our aim is to develop a comprehensive standard of care for these patients, facilitated by a collaborative approach between cardiology and immunology, to predict and potentially mitigate severe immunological sequelae. The primary objective is to calculate the prevalence of lymphopenia in patients with conotruncal cardiac defects who underwent open-heart surgery with thymectomy. We then tracked the trend of lymphocyte count over a couple of years after the surgery and identified possible factors and correlated it with the infection rate.

## Method

### Study design

This is a retrospective cohort study conducted at King Abdulaziz University from January 2017 and December 2022.

### Population and sampling

The study’s eligible patients were limited; therefore, a total sampling technique was employed. All patients who had their first heart surgery with thymectomy under the age of 2 years at King Abdulaziz University Hospital between January 2017 and December 2022 were included. Patients’ lists were gathered from the hospital information system based on ICD-10 codes for conotruncal cardiac abnormalities. It included Double Outlet Right Ventricle, Transposition of the great vessels, Tetralogy of Fallot, Truncus arteriosus, and other complex heart defects. All patients in the cohort underwent complete thymectomy as part of the standard surgical approach following median sternotomy. There was no variation in the extent of thymic resection across the study population, and partial thymic preservation was not performed in any case. Lists were filtered for duplicates and non-eligible patients.

### Procedure

**Fig. 1:**
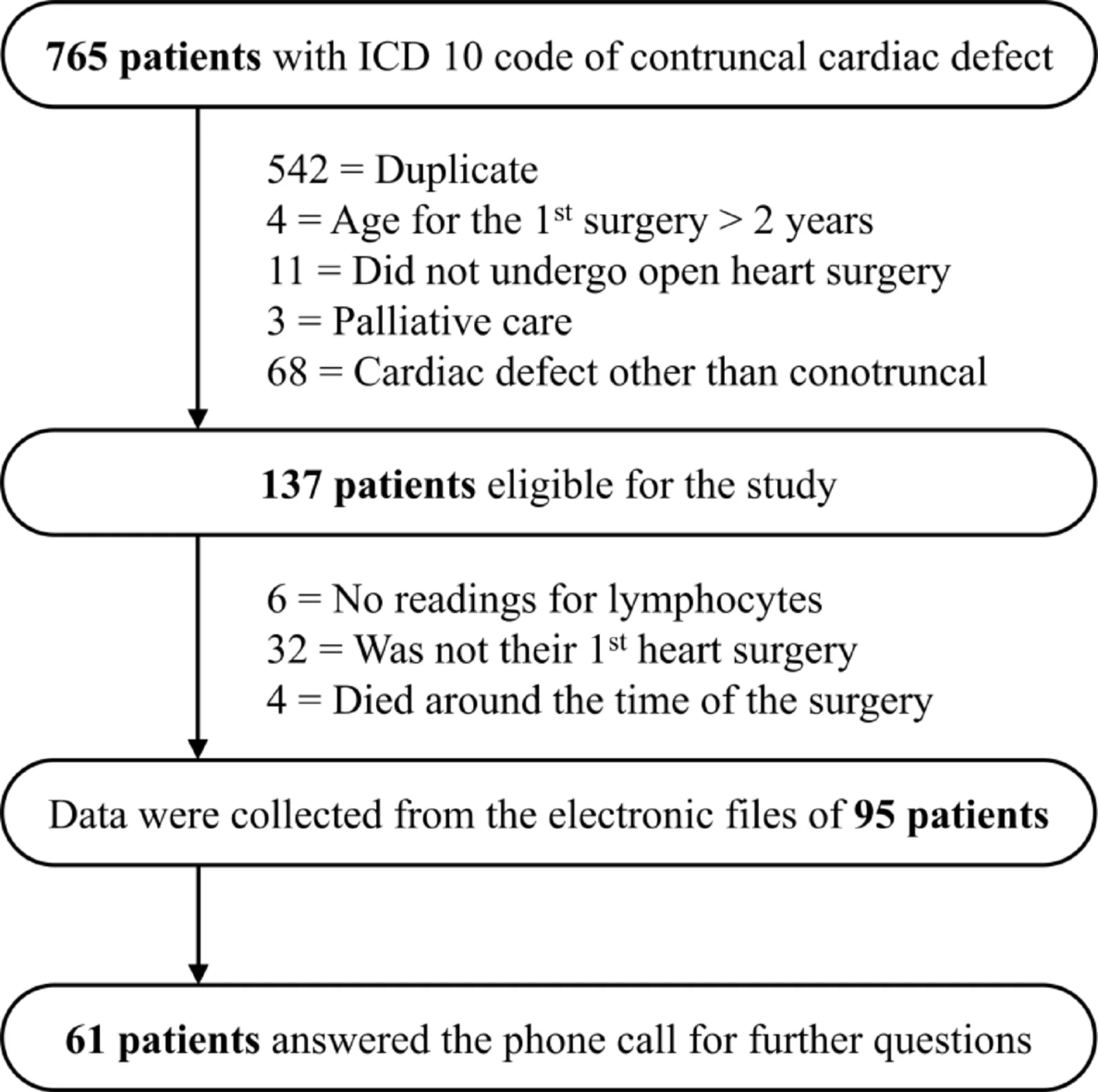
Flow chart depicting the sampling process

After getting the patient lists from the hospital’s medical records department, well-trained senior medical students did the filtration process according to the eligibility criteria. [Figure 1] Relying on the hospital electronic file system, data collection sheets were filled with the accessible data. Frequent monitoring by the principal investigator was conducted to ensure data accuracy and reliability. Parents were subsequently contacted by telephone to verify previously collected data and obtain updated information regarding the patient’s current health status, laboratory investigations performed outside our institution, hospital admissions, and emergency department visits related to infections.

### Variables and outcomes

The data collection sheet included a section for the patient’s personal data and anthropometric measurements. The second section aimed to explore the details of the cardiac defect and the surgery, followed by a section on postoperative information and consequences. Lastly, a section for pre- and post-operative laboratory results. All fillable fields were either multiple-choice or numerical. [Appendix 1: the data collection sheet].

The main outcome for the study was to calculate the prevalence of lymphopenia in patients with conotruncal cardiac defects who underwent open heart surgery with thymectomy over the first week post operative. The secondary outcomes were the trend of lymphocyte count 1 month after surgery and at age of 2–5 year. Also, we calculated the frequency of simple and sever infections per year. It was calculated as an average of 3 years following the surgery. Simple infection defined as any viral or bacterial illness that did not require a hospital admission or full course of intravenous antibiotic. Otherwise, sever infection defined as any viral, fungal or bacterial illness necessitates a hospital or intensive care admission, or a full course of intravenous antimicrobial therapy. Age specific Lymphocyte reference range and cutoff for lymphopenia were based on those provided by Severn Pathology (North Bristol NHS Trust) for the full blood count [20]. 

### Data management and statistical analysis

Descriptive statistics were used to summarize the cohort characteristics. Categorical variables were presented as frequencies and percentages, and continuous variables were presented as medinals with interquartile ranges (IQR). Comparisons between patients with and without post-operative lymphopenia were performed using Pearson’s chi-square test for categorical variables and the Wilcoxon rank-sum test for continuous variables, as appropriate. The relationship between pre-operative lymphocyte count and the difference between pre- and post-operative lymphocyte count was visually explored and quantified. The distribution of the pre-to-post-operative change in lymphocyte count and its relationship with the baseline lymphocyte count, shown with an illustrative fitted line, were displayed graphically.

A multiple linear regression model was constructed to assess the association between pre-operative lymphopenia and the absolute post-operative lymphocyte count, expressed as Beta coefficients and standard error (SE). Given that the definition of lymphopenia is age-dependent, age itself was not included as a covariate in the models to avoid collinearity. The models were adjusted for failure to thrive, selected a priori as a marker of nutritional status given the established association between malnutrition, thymic atrophy, and lymphopenia. A two-sided p-value of < 0.05 was considered statistically significant. All analyses were conducted using SAS version 9.4.

### Ethical considerations

Ethical approval was obtained from the Unit of Biomedical Ethics at King Abdulaziz University. As interviews were conducted via telephone, verbal informed consent was obtained from participants’ representatives prior to participation, in accordance with the ethics committee-approved protocol. Participants were informed about the study objectives, voluntary participation, and confidentiality of their information.

## Results

### Sample demographic at the time of open-heart surgery

The data were collected from 95 patients. The median age at the time of the first open-heart surgery 9.0 (IQR 5.0, 12.0) months, with approximately 57% being over 6 months of age. The median weight at the time of the surgery was 5.0 (1.0, 25.0) Kg, where 41 (43.15%) babies plotted below the 3rd percentile in the corresponding growth chart. Male patients constituted 63.16%. Tetralogy of Fallot (TOF) represented the highest proportion among the cardiac defects with 51 patients (53.68%), followed by Transposition of the great vessels (TGA) (20, 21.05%), DORV (17, 17.89%), and complex heart defects (7, 7.37%). The majority (73.68%) had a successful complete repair, with subsequent chylothorax being reported in 5 (3.5%) patients. The average incidence of both simple and severe infection was 0.6 per year over 3 years following the surgery. [Table 1]

**Table 1:** Baseline demographic and clinical characteristics of patients according to the presence of postoperative lymphopenia

Variables [Median (Q1, Q3) | *N* (%)]		Total	Lymphopenia within one week postoperatively		*P*- value
		[*n* = 95]	No [*n* = 43]	Yes [*n* = 52]	
Age at the surgery time (months)		9.0 (5.0, 12.0)	12.5 (7.0, 19.0)	6.0 (4.0, 12.0)	< 0.001
Weight at the surgery time (Kg)		6.7 (4.7, 8.5)	7.5 (6.0, 10.0)	5.6 (4.1, 7.7)	0.001
Weight percentile at the surgery time (%)		5.0 (1.0, 25.0)	10.0 (1.0, 37.0)	2.0 (1.0, 17.0)	0.071
Baseline lymphocytes count preoperative (×10^9/L)		6.4 (4.8, 9.0)	7.2 (5.4, 9.3)	5.8 (4.1, 8.5)	0.083
Lymphocyte count one month postoperatively (×10^9/L)		3.9 (2.8, 5.3)	5.2 (3.4, 6.7)	3.4 (2.1, 4.8)	0.006
Lymphocyte count at 2–5-year-old (×10^9/L)		3.0 (2.3, 3.9)	3.2 (2.5, 4.0)	3.0 (2.2, 3.7)	0.789
Gender	Female	35 (36.8)	15 (35.7)	20 (37.7)	0.839
	Male	60 (63.2)	27 (64.3)	33 (62.3)	
Cardiac defect	DORV	17 (17.9)	4 (9.5)	13 (24.5)	0.167
	TGA	20 (21.1)	11 (26.2)	9 (17.0)	
	TOF	51 (53.7)	25 (59.5)	26 (49.1)	
	Complex	7 (7.4)	2 (4.8)	5 (9.4)	
Syndrome	Not syndromic	80 (84.2)	37 (88.1)	43 (81.1)	0.614
	Down’s	8 (8.4)	2 (4.8)	6 (11.3)	
	DiGeorge	3 (3.2)	1 (2.4)	2 (3.8)	
	CHARGE	1 (1.1)	0 (0)	1 (1.9)	
	Holt Oram	1 (1.1)	1 (2.4)	0 (0)	
	Noonan’s	2 (2.1)	1 (2.4)	1 (1.9)	
Operation outcome	Successful complete repair	70 (73.7)	31 (73.8)	39 (73.6)	0.999
	Successful partial repair	16 (16.8)	7 (16.7)	9 (17.0)	
	Successful with consequences	9 (9.5)	4 (9.5)	5 (9.4)	
Age at surgery time	≤ 6-month-old	41 (43.2)	10 (23.8)	31 (58.5)	< 0.001
	> 6-month-old	54 (56.8)	32 (76.2)	22 (41.5)	
Failure to thrive	No	54 (56.8)	28 (66.7)	26 (49.1)	0.085
	Yes	41 (43.2)	14 (33.3)	27 (50.9)	
Anti-failure medication	No	14 (18.9)	6 (20.7)	8 (17.8)	0.584
	Temporary (≤ 3 months postoperatively)	37 (50.0)	16 (55.2)	21 (46.7)	
	Chronic (> 3 months postoperatively)	23 (31.1)	7 (24.1)	16 (35.6)	
Baseline lymphopenia	No	84 (88.4)	42 (100)	42 (79.2)	0.001
	Yes	11 (11.6)	0 (0)	11 (20.8)	
Lymphopenia one month postoperatively	No	24 (57.1)	12 (85.7)	12 (42.9)	0.008
	Yes	18 (42.9)	2 (14.3)	16 (57.1)	
Lymphopenia at the age of 2–5 years of age	No	23 (95.8)	5 (83.3)	18 (100)	0.076
	Yes	1 (4.2)	1 (16.7)	0 (0)	
Antibiotic therapy for 3 months postoperatively	No	62 (89.9)	23 (92.0)	39 (88.6)	0.656
	Yes	7 (10.1)	2 (8.0)	5 (11.4)	
Episodes of simple infection per year (for 3 years postoperatively)	0	30 (58.8)	14 (70.0)	16 (51.6)	0.308
	1	15 (29.4)	5 (25.0)	10 (32.3)	
	2	2 (3.9)	1 (5.0)	1 (3.2)	
	3	4 (7.8)	0 (0)	4 (12.9)	
Episodes of severe infection per year (for 3 years postoperatively)	0	43 (89.6)	17 (89.5)	26 (89.7)	0.39
	1	4 (8.3)	1 (5.3)	3 (10.3)	
	2	1 (2.1)	1 (5.3)	0 (0)	

DORV, double outlet right ventricle; TGA, transposition of the great arteries; TOF, tetralogy of Fallot

### Lymphocyte trend

The median baseline lymphocyte count was 6.4 (4.8, 9.0) ×10^9/L. The median count declined to 3.1 (2.3, 4.2) within the first week of post-open-heart surgery with thymectomy. Later median lymphocyte trends were 3.9 (2.8, 5.3) after the first month of surgery and 3.0 (2.3, 3.9) between 2 and 5 years of age.

The proportion of patients who had baseline lymphopenia was 11/93 (11.6%). Within 1 week following the surgery, the proportion increased to 52/95 (54.7%). The prevalence was 18/42 (42.9%) one month following the surgery and 1/23 (4.2%) at 2–5 years of age, respectively.

### Factors contributing to lymphopenia

**Fig. 2:**
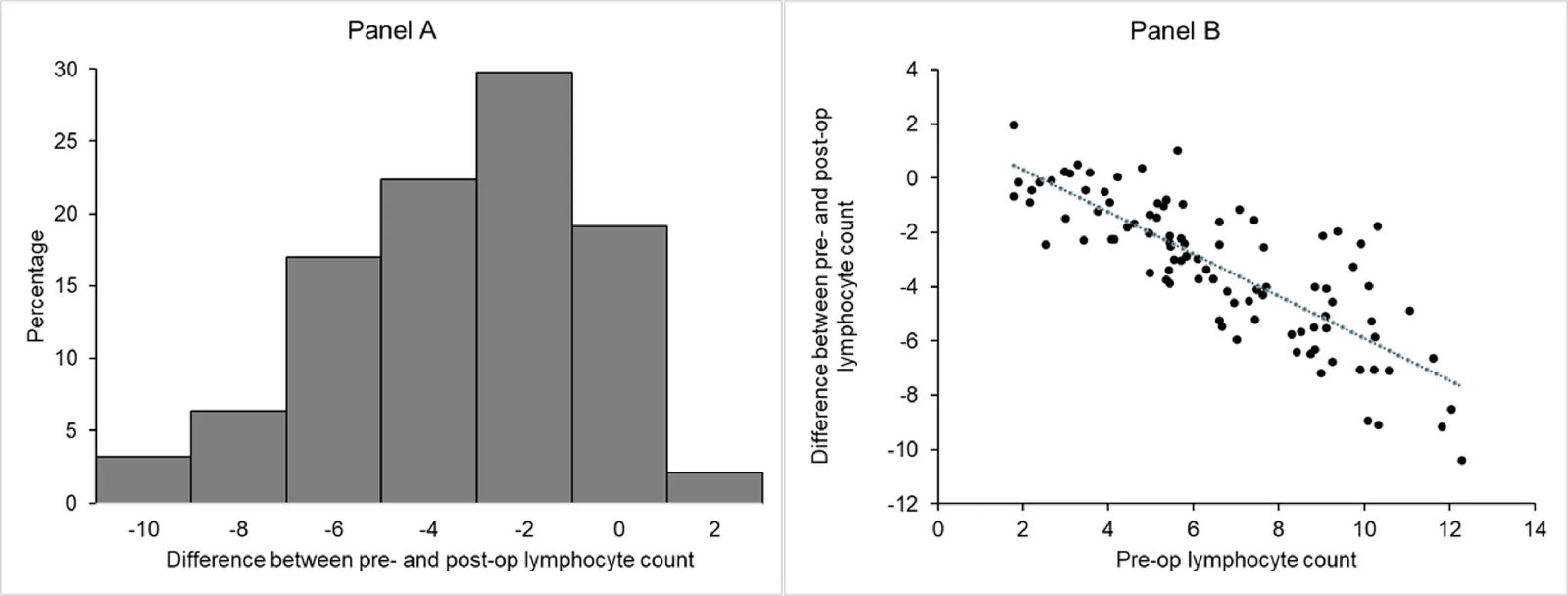
**(A)** Distribution of the the difference between pre- and post-operative lymphocyte count. **(B)** Scatter plot and linear regression line demonstrating the relationnhip between pre-operative lymphocyte count and the difference between pre- and post-operative lymphocyte count

In the bivariate analysis, the median age of children who developed postoperative lymphopenia was 6.0 months (IQR 4.0–12.0), compared to 12.5 months (IQR 7.0–19.0) in those who did not (*P* = 0.0001). A lower median weight at the time of surgery was also associated with a higher likelihood of developing postoperative lymphopenia [5.6 kg (IQR 4.1–7.7) vs. 7.5 kg (IQR 6.0–10.0); *P* = 0.001]. All children who had lymphopenia at baseline (11/11) remained lymphopenic postoperatively, compared to 42/84 (50%) of those who were not lymphopenic at baseline (*P* = 0.001). The distribution of the change in lymphocyte count and its relationship with the baseline count are shown in Fig. 2A and B, respectively, with larger declines observed at higher baseline counts. Among patients who were lymphopenic postoperatively, 16/52 remained lymphopenic beyond the first postoperative month, while 12/52 recovered from lymphopenia (*P* = 0.008). Follow-up lymphocyte counts beyond one month were unavailable for the remaining 24 and 29 patients in the baseline lymphopenia and non-lymphopenia groups, respectively.

In multivariable analysis, being lymphopenic at baseline was associated with a decrement in lymphocyte count postoperatively by 1.2 × 10^9/L (*P* = 0.02). This association remained significant after adjustment for being failure to thrive (*P* = 0.04). Being failure to thrive by itself was an independent predictor for a decrement in lymphocyte count after adjustment for a baseline lymphopenia [β = -0.67 ± 0.33 (*P* = 0.04)]. [Table 2].

**Table 2:** Multiple linear regression of the association between pre-operative lymphopenia and post-operative lymphocyte count (*n* = 95)

Variables	Unadjusted model		Adjusted model	
	Beta ± SE	*P*-value	Beta ± SE	*P*-value
Lymphopenia	-1.20 ± 0.51	0.020	-1.05 ± 0.50	0.041
Failure to thrive	-0.77 ± 0.33	0.022	-0.67 ± 0.33	0.044

### Infection rate and the necessity of antibiotic therapy

The use of antibiotics throughout the 3 months postoperative was comparable between patients with and without early postoperative lymphopenia (11.4% vs. 8.0%, *p* = 0.656). Annual frequency of simple infections during the first 3 postoperative years did not differ significantly between the two groups (*p* = 0.308). The incidence of severe infections during the same follow-up period was low and comparable between groups (*p* = 0.390), with approximately 90% of patients in both groups reporting no severe infections.

## Discussion

We investigated the incidence of lymphopenia and its predictors in infants with congenital heart disease underwent surgical repair. Complete thymectomy is a typical approach in such operations for better visualization. We utilized the total lymphocyte count in ×10^9/L to report lymphopenia according to the age specific cutoff, others, used different biomarkers including lymphocyte subpopulation (CD3⁺, naïve CD4⁺ and CD8⁺) and T-cell Receptor Excision Circles (TRECs) [12, 14, 18, 21]. Although lymphocyte subsets and receptors analysis appears to be more specific, absolute total lymphocyte count is a sensitive and reliable screening marker for the immune state [22]. 

The prevalence of an age specific lymphopenia in our cohort was 54.7%. Prevalence is variable in literature depending on many variables including the population age, screening marker, type of congenital heart disease and associated syndromes [11, 18, 19, 21]. It is reported to be as high as 100% in neonates post cardiac surgery and thymectomy [12]. Moreover, a significant decrement in lymphocyte counts and other marker is observed even not crossing the lymphopenia cutoff [18, 21].

Younger age and lower weight at the time of surgery and baseline lymphopenia were associated with post-operative lymphopenia, whereas failure to thrive was associated with a greater decrement in the post-operative lymphocyte count. The postoperative reduction in lymphocyte count was not confined to infants with low baseline counts; as shown in Table 1, lymphopenia prevalence rose from 11.6% (11/95) preoperatively to 54.7% (52/95) within the first postoperative week, indicating a surgery-associated decline across the cohort rather than persistence of pre-existing low counts alone. Consistently, earlier age of surgery and complete thymectomy were the strongest predictors observed in literature [11, 12, 14, 18, 21]. Interestingly, pre-operative lymphopenia is common in children with CHD and associated with worse post-operative outcomes and higher mortality rate [13]. In more specific populations, Fontan physiology, lymphatic dysfunction, protein-losing enteropathy, being 22q11.2 deletion syndrome and persistent SpO₂ <95% have been reported as risk factors associated with these adverse outcomes [14, 19]. 

Small fraction of our population had a lymphocyte count reading years after surgery, hence, the fate of the lymphopenia cannot be reliably reported. Studies showed that low lymphocyte count and other markers were persistently low for years after surgery [11, 12, 18, 21], whereas, some reports suggest that a non-syndromic CHD might have a transient course and improvement overtime is warranted [14]. differently, Fontan patients who have other comorbidities such as lymphatics failure may have a progressive lymphopenia over the years that was attributed to the hypoxic environment for a developing thymus [19]. Preservation of some of the thymus tissue during the surgery is associated with an improvement in the lymphocyte count and other immunological markers over time [11, 12, 23–25]. Notably, B-cell count was the least affected [23, 25]. 

Our data suggest that baseline and persistent lymphopenia were not associated with increase rate of mild or sever infection in the following years. This goes in align with literature [12], however, some studies report an increase in viral illness and delayed viral clearance [12, 19, 26]. Importantly, the immune consequences of early thymectomy extend well beyond absolute lymphocyte counts [10]. demonstrated that children who underwent complete thymectomy during CHD repair developed a consistent immunophenotype characterized by markedly low naïve CD4⁺ T cells and an inverted naïve-to-memory ratio, closely mirroring the profile seen in 22q11.2 deletion syndrome, despite near-normal total T cell counts in some cases. Their clinical presentations ranged from recurrent EBV infection with hepatic fibrosis to immune cytopenias, underscoring that total lymphocyte count alone may substantially underestimate the degree of functional immune compromise in this population [10]. 

Longterm lymphopenia is attributed to the halt of hemopoiesis during an immune developing window, leading to irreversible depletion of naïve T cells [12], and ultimately, premature aging and immunosenescence [11, 21]. Peripheral T-cell expansion and preserved memory T-cell function compensate for reduced thymic output [11, 18]. In other situations where lymphatics malformation coexist with CHD, chronic lymphatic congestion causes continuous lymphocyte and immunoglobulin loss independent of thymic output [19]. 

In light of the present results, we recommend a longitudinal follow up for the immune function in CHD patients primarily by checking the total lymphocyte counts, together with clinical surveillance. Pre-operative risk stratification based on lymphocyte count and other immunological markers is also recommended. From surgical perspective, preserving some thymic tissue may contribute to a better immunological outcome.

Some limitations must be highlighted. The retrospective nature of the study restrained the investigators from gathering as much information as required, including different lymphocyte subpopulations, B-cell count, response to immunization, and long-term lymphocyte counts. Also, most of the patients were lost to follow-up less than 1 year post-operatively; hence, many clinical outcomes and infection episodes were unable to be traced. In addition, our database did not include all potential variables that could be associated with lymphopenia (like cardiopulmonary bypass time, aortic cross-clamping, postoperative sepsis, and the use of corticosteroids). A comparable control group would add much value to the reported results. Long term outcomes (including infections) are limited due to high attrition rate and the nature of data collection. A comparable control group would add much value to the reported results. Notably, patients with 22q11.2 deletion syndrome were included in the cohort, as they represent an inherent component of the conotruncal population in clinical practice. While this reflects real-world heterogeneity, it introduces a layer of immune vulnerability independent of thymectomy. Subgroup analysis separating syndromic from non-syndromic patients was limited by sample size and is recommended as a priority in future larger studies.

## Conclusion

Immediate and long-term lymphopenia is highly prevalent post CHD surgical repair. Earlier age at the time of surgery, baseline lymphopenia, and being failure to thrive were significant predictors for lymphopenia post CHD surgical repair with total thymectomy. Subsequent lymphopenia not necessarily correlates with increase rate or severity of the infections. Pre-, post-operative and long-term lymphocyte count screening and clinical monitoring are warranted.

## Supplementary Information

Supplementary material 1.

## Data Availability

No datasets were generated or analysed during the current study.
