# Supplementary material for: Lymphopenia in thymectomized infants following congenital heart disease repair: implications for protective measures
Source: J Cardiothorac Surg. 2026 Aug 17;21:887. doi: 10.1186/s13019-026-04602-y (PMC13760879; doi:10.1186/s13019-026-04602-y)
Supplement: Supplementary file 1 [file 13019_2026_4602_MOESM1_ESM.docx]

[Appendix 1] Data collection sheet

Immune Function in Thymectomiezed Infants Post Cardiac Correction Surgery

| **Demographic data** | | | | | | | | | | | | | | | | | |
| --- | --- | --- | --- | --- | --- | --- | --- | --- | --- | --- | --- | --- | --- | --- | --- | --- | --- |
| **Contact No.** | | | |  | | | | **ID No.** | | | | |  | | | | |
| **MRN** (VIDA) | | | |  | | | | **MRN** (Pheonix) | | | | |  | | | | |
| **Age** at the time of surgical correction (months) | | | |  | | | | **Gender** | | | | | ◯ Female ◯ Male | | | | |
| **Weight** at the time of surgical correction (kg) | | | |  | | | | **Weight** **percentile** according to the Saudi growth chart | | | | |  | | | | |
| **Height** at the time of surgical correction (cm) | | | |  | | | | **Height** **percentile** according to the Saudi growth chart | | | | |  | | | | |
|  | | | | | | | | | | | | | | | | | |
| **Cardiac condition details** | | | | | | | | | | | | | | | | | |
| **Type of Cono-truncal defect**  __________________ | | | ◯ TGA  ◯ Truncus Arteriosus  ◯ TOF  ◯ DORV  ◯ Ebstein anomaly | | | | | | **Has the child ever diagnosed with any of the following?**  __________________ | | | | | ◯ Malignancy  ◯ Immunodeficiency  ◯ Allergy  ◯ Failure to thrive  ◯ Autoimmune  ◯ Rheumatological | | | |
| **OR details**, complications, type of surgery | | |  | | | | | | **Other diseases/syndromes** | | | | |  | | | |
|  | | | | | | | | | | | | | | | | | |
| **Post-operative details** | | | | | | | | | | | | | | | | | |
| **OR outcome** | | ◯ Successful complete repair  ◯ Successful partial repair  ◯ Successful with consequences  ◯ Failed | | | | | | | | | **Thymectomy done during OR** | | | | | ◯ Yes  ◯ No | |
| **Anti-failure medications** | | ◯ Temporary (3 months post Op)  ◯ Chronic (>3 months post Op)  ◯ No | | | | | | | **Prophylactic antibiotic** | | ◯ Temporary (3 months post Op)  ◯ Chronic (>3 months post Op)  ◯ No | | | | | | |
| **Other medications** | | ◯ Regular Immunoglobulin  ◯ Steroid therapy  ◯ Biological agent | | | | | | |  | | | | | | | | |
| **Episodes** of hospital admissions/year because of **simple** infections after the OR (for the 3 years following the OR) | | | | | |  | | | **Episodes** of hospital admissions/year because of **severe** infections after the OR (for the 3 years following the OR) | | | | | | | |  |
|  | | | | | | | | | | | | | | | | | |
| **Labs** | | | | | | | | | | | | | | | | | |
|  | Baseline labs before the thoracotomy | | | | Labs 2-7 days after the thoracotomy | | | | | Labs 4+ weeks after the thoracotomy | | | | | Labs at the age of 2-5 years of age | | |
| **Total WBC** |  | | | |  | | | | |  | | | | |  | | |
| **Lymphocyte** |  | | | |  | | | | |  | | | | |  | | |
| **Neutrophil** |  | | | |  | | | | |  | | | | |  | | |
| **Platelet count** |  | | | |  | | | | |  | | | | |  | | |
| **Hemoglobin** |  | | | |  | | | | |  | | | | |  | | |
| **MCV** |  | | | |  | | | | |  | | | | |  | | |
| **MCH** |  | | | |  | | | | |  | | | | |  | | |
| **HCT** |  | | | |  | | | | |  | | | | |  | | |
|  | | | | | | | | | | | | | | | | | |
| **Has the child had this workup done outside?** | ◯ Karyotype  ◯ Whole Exome Sequencing  ◯ Lymphocyte subset (Flowcytometry) | | | | | | ◯ Immunoglobulin levels  ◯ Diphtheria/tetanus Ig response  ◯ Lymphocyte proliferation or transformation studies | | | | | ◯ CGD testing/DHR/burst test  ◯ Complements  ◯ Cytokines (IL, JAK ..) | | | | | |
